# PCBP1 regulates LIFR through FAM3C to maintain breast cancer stem cell self-renewal and invasiveness

**DOI:** 10.1080/15384047.2023.2271638

**Published:** 2023-11-06

**Authors:** William S. Streitfeld, Annamarie C. Dalton, Breege V. Howley, Philip H. Howe

**Affiliations:** Department of Biochemistry and Molecular Biology, Medical University of South Carolina, Charleston, SC, USA

**Keywords:** Breast cancer, epithelial-mesenchymal transition (EMT), gene regulation, invasion, migration, molecular cell biology, receptor regulation, signal transduction, STAT3, stem cells

## Abstract

The poly(rC) binding protein 1 gene (PCBP1) encodes the heterogeneous nuclear ribonucleoprotein E1 (hnRNPE1), a nucleic acid-binding protein that plays a tumor-suppressive role in the mammary epithelium by regulating phenotypic plasticity and cell fate. Following the loss of PCBP1 function, the FAM3C gene (encoding the Interleukin-like EMT inducer, or “ILEI” protein) and the leukemia inhibitory factor receptor (LIFR) gene are upregulated. Interaction between FAM3C and LIFR in the extracellular space induces phosphorylation of signal transducer and activator of transcription 3 (pSTAT3). Overexpression and/or hyperactivity of STAT3 has been detected in 40% of breast cancer cases and is associated with a poor prognosis. Herein, we characterize feed-forward regulation of LIFR expression in response to FAM3C/LIFR/STAT3 signaling in mammary epithelial cells. We show that PCBP1 upregulates LIFR transcription through activity at the LIFR promoter, and that FAM3C participates in transcriptional regulation of LIFR. Additionally, our bioinformatic analysis reveals a signature of transcriptional regulation associated with FAM3C/LIFR interaction and identifies the TWIST1 transcription factor as a downstream effector that participates in the maintenance of LIFR expression. Finally, we characterize the effect of LIFR expression in cell-based experiments that demonstrate the promotion of invasion, migration, and self-renewal of breast cancer stem cells (BCSCs), consistent with previous studies linking LIFR expression to tumor initiation and metastasis in mammary epithelial cells.

## Introduction

Mammary carcinoma is a leading cause of death among women in the US, second only to heart disease.^1^ Patients diagnosed with metastatic breast cancer have a five-year survival rate of less than 25%.^2^ The high prevalence of breast cancer in women in the US is a major public health concern and highlights the need to identify targetable biochemical mechanisms.^3^ Furthermore, the high mortality rate of patients diagnosed with metastatic mammary carcinoma prioritizes research aimed at understanding the cellular events involved in the pathology of advanced disease.

PCBP1 encodes a nucleic acid-binding protein that regulates cellular events in both the nucleus and the cytoplasm. Our previous studies have shown that multiple intracellular functions of PCBP1 participate in carcinoma progression.^4–6^ Studies by our group and others have shown that PCBP1 suppresses epithelial-to-mesenchymal transition (EMT) in mammary epithelium and that it also acts as a master regulator of epithelial cell polarity and differentiation status.^5,7–10^ Loss of PCBP1 function in normal murine mammary gland (NMuMG) cells confers an invasive, migratory phenotype that initiates tumors. Additionally, NMuMG cells with loss of PCBP1 function gain stemness potential and differentiation potency, as demonstrated by full reconstruction of the mouse mammary gland following injection into surgically cleared fat pads.^7^ The PCBP1-driven characteristics of disease in our mouse model demonstrate that EMT manifests in neoplastic cells that exemplify the characteristics of breast cancer stem cells (BCSCs).^7,11^

Our lab has previously characterized a PCBP1-dependent mechanism of tumor suppression that involves the inhibition of mRNA translation. Loss of PCBP1 expression or phosphorylation of PCBP1 by AKT2 kinase results in a loss of binding between PCBP1 and conserved 3’ mRNA secondary structures, resulting in increased protein translation. FAM3C has been shown to be among the mRNAs that contain the 3’ element, revealing links between PCBP1 expression, FAM3C expression, phenotypic changes, and carcinoma progression.^5,7,8^ Following PCBP1 knockdown, NMuMG cells upregulate FAM3C and display EMT.^8^ Additionally, knockdown of FAM3C expression has been shown to attenuate TGF-β-mediated EMT.^7,12^ Most recently, it was shown that knockdown of PCBP1 coincides with upregulation of the leukemia inhibitory factor receptor (LIFR) gene.^7^ LIFR expression and signaling have been shown to promote dormant cancer, chemoresistance, tumorigenesis, metastasis, and BCSC phenotypes in preclinical mammary carcinoma studies.^7,13,14^ Our previous study also demonstrated an interaction between FAM3C and LIFR, with the ensuing phosphorylation of STAT3.^7^ Upregulation of LIFR expression in mammary epithelial cells increases STAT3 activation via Y705 phosphorylation.^7,15^ Notably, orthotopic grafts of NMuMG cells with PCBP1 knockdown cause mammary tumors and metastases in the lungs of immunocompromised mice, and knockdown of either FAM3C or LIFR in the same cells attenuates tumor growth and metastatic burden.^7^

Constitutive STAT3 activation has been detected in multiple cancer types, including in 40% of breast cancer cases.^16^ Recent evidence has demonstrated that STAT3 overexpression and/or hyperactivity play a critical role in triple-negative breast cancer (TNBC) pathology, and that STAT3 signaling mechanisms contribute to chemoresistance in TNBC.^16,17^ Moreover, STAT3 is required for normal ductal development in the mammary gland and is sufficient for maintaining murine embryonic stem cell pluripotency.^18–20^ Recent studies have suggested that STAT3 signaling contributes to phenotypic changes associated with BCSCs through its regulation of downstream effectors, including the TWIST family of transcription factors.^21,22^

Given the role of PCBP1 in our mouse model of mammary carcinoma and the increase in FAM3C and LIFR expression following loss of PCBP1, we sought to identify the mechanism(s) governing LIFR expression in mammary epithelium. Herein, we demonstrate that regulation of LIFR occurs at the transcriptional level and show that FAM3C is involved in the modulation of LIFR protein expression. Additionally, we demonstrate how changes in LIFR signaling influence downstream regulation of gene expression. Experimental data describe the regulation of transcriptional activation at the LIFR promoter locus and indicate that the loss of PCBP1 expression induces FAM3C/LIFR-driven signaling that regulates transcriptional events associated with carcinoma pathology. Additionally, we show that modulation of LIFR signaling affects the expression of TWIST1 and that TWIST1 participates in the regulation of LIFR through a reciprocal mechanism. Finally, we show that modulation of LIFR expression directly affects invasiveness and self-renewal in NMuMG cells following loss of PCBP1 function.

## Results

### Loss of PCBP1 expression upregulates LIFR

**Figure 1.**
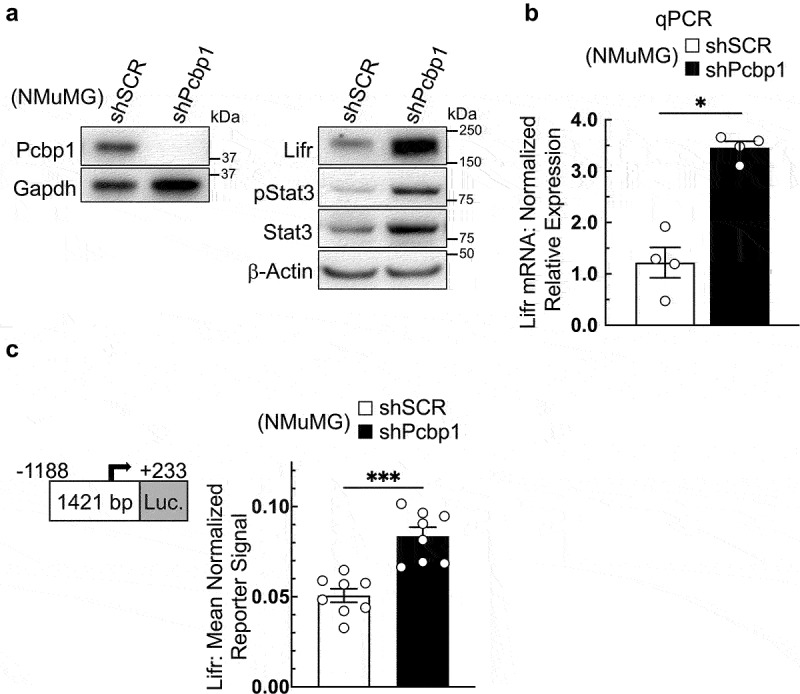
Loss of PCBP1 expression upregulates LIFR. (a) Immunoblot analysis of NMuMG cells transduced either with shRNA containing “scrambled” non-targeting control (shSCR) or shRNA targeting PCBP1 (shPcbp1). (b) qPCR analysis of the cells used in panel “a”. (c) dual luciferase reporter assay comparing the cells used in panel “a”, using the indicated region of the mouse LIFR proximal promoter upstream of a firefly luciferase open reading frame. All data points represent independent experiments, each performed in triplicate. Error bars represent standard error of the mean, *P<.05, ***P<.001 (unpaired Mann-Whitney U test).

Following knockdown of PCBP1 in NMuMG cells, we observed an increase in LIFR expression and a concomitant increase in STAT3 phosphorylation at Y705 (pSTAT3). Immunoblot analysis demonstrated an increase in LIFR protein expression in NMuMG shPCBP1 cells (hereafter “shPCBP1 cells”) relative to control cells transduced with “scrambled” nonspecific shRNA (hereafter “shSCR cells”) (Figure 1a). To determine whether LIFR upregulation occurred due to an increase in transcription, LIFR mRNA levels were measured by quantitative real-time PCR (qPCR). Upregulation of LIFR mRNA was greater than three-fold in shPCBP1 cells relative to control cells (Figure 1b). To determine whether the increase in LIFR mRNA resulted from an increase in transcription initiation, a DNA sequence flanking the LIFR proximal promoter was isolated from the genomic DNA of NMuMG cells and inserted into a plasmid vector upstream of a firefly luciferase open reading frame (ORF). Transfection of the plasmid vector followed by a dual-luciferase assay revealed an increase in the reporter signal in shPCBP1 cells relative to control cells (Figure 1c). Comparison of LIFR mRNA degradation rates following PCBP1 knockdown did not provide any evidence suggesting that an increase in mRNA stability contributed to the observed increase in LIFR mRNA (data not shown). These results show that a loss of PCBP1 expression in NMuMG cells results in increased LIFR expression through a mechanism involving increased transcriptional activity at the LIFR promoter.

### FAM3C participates in regulation of LIFR expression

**Figure 2.**
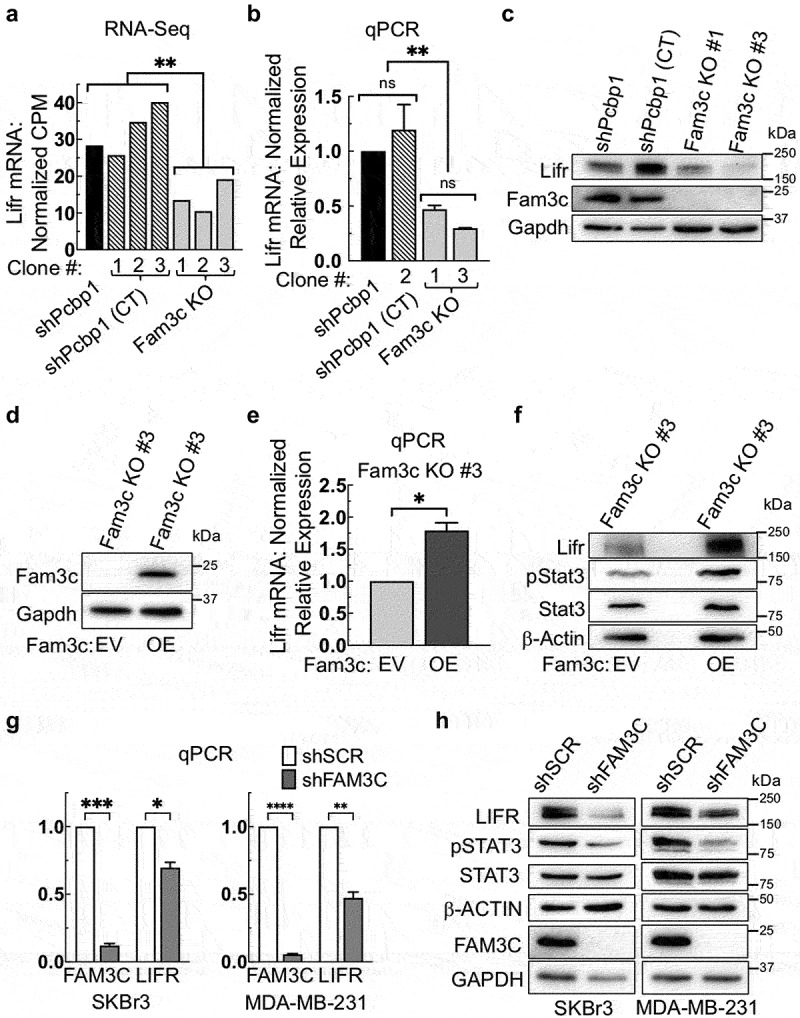
FAM3C participates in regulation of LIFR expression. (a) RNA-Seq analysis of total RNA isolates from the indicated NMuMG derivative cell lines; “CT” (control) indicates additional shPCBP1 cell lines (“clones”) treated with Cas9 in the absence of gene-specific guide RNAs. Bars represent normalized counts per million paired-end reads from one RNA sample per cell line tested, **q < 0.01 (DESeq FDR step-up). (b) qPCR validation of the RNA-Seq data in panel “a”. Error bars represent SEM from three independent experiments, each performed in triplicate, *P < .01 (one-way ANOVA). (c) Immunoblot analysis of the cells shown in panel “b”. (d) Immunoblot analysis of FAM3C KO cells shown in panel “a” following transduction with lentivirus containing either empty vector (EV) or a vector for overexpression rescue (OE) of mouse FAM3C protein. (e) qPCR analysis of the cells used in panel “d”. Error bars represent SEM from duplicate independent experiments, each performed in triplicate, *P < .05 (unpaired Student’s *t*-test). (f) Immunoblot analysis of the cells used in panel “d” using the indicated antibodies. (g) qPCR analysis using SKBr3 and MDA-MB-231 human breast cancer cell lines transduced either with “scrambled” non-targeting control shRNA (shSCR) or shRNA targeting FAM3C (shFAM3C). Error bars represent SEM from duplicate independent experiments, each performed in triplicate, *P < .05, **P < .01, ***P < .001, ****P < .0001 (unpaired Student’s *t*-tests). (h) Immunoblot analysis using the cells shown in panel “g”. ns, not significant; SEM, standard error of the mean.

To further characterize the landscape of FAM3C/LIFR-dependent gene regulation, FAM3C was knocked out (KO) in shPCBP1 cells using CRISPR-Cas9 (hereafter “FAM3C KO cells”). Total pooled RNA isolates were sequenced (RNA-Seq) and differentially expressed genes (DEGs) were analyzed. Comparisons were made between shPCBP1 cells, multiple FAM3C KO cell line “clones,” and multiple “control” (CT) cell line clones (hereafter “shPCBP1 CT cells”). shPCBP1 CT cells were created by treating cells with Cas9 in the absence of gene-specific guide RNAs. Interestingly, one of the genes found to be differentially expressed following FAM3C KO was LIFR (Figure 2a). To validate the RNA-Seq results, a subset of cell line clones was selected for further study and qPCR was performed. qPCR results also demonstrated a loss of LIFR mRNA following FAM3C KO (Figure 2b). Immunoblot analysis showed that the loss of LIFR mRNA was sufficient to cause loss of LIFR protein expression (Figure 2c). To determine whether the loss of LIFR expression could be restored by re-expressing FAM3C, one of the FAM3C KO cell lines was selected for further study and transduced with either an “empty vector” (EV) control plasmid or a vector containing the mouse FAM3C ORF. Following the confirmation of stable overexpression (OE) of FAM3C (hereafter “FAM3C KO OE cells”) (Figure 2d), qPCR analysis was performed, and the results showed that LIFR mRNA was increased relative to control cells lacking FAM3C (Figure 2e). Immunoblot analysis showed that the restoration of FAM3C expression was sufficient to measurably increase abundance of LIFR protein and concomitant levels of pSTAT3 relative to cells lacking FAM3C (Figure 2f). These data suggest that FAM3C directly participates in the regulation of LIFR in shPCBP1 cells. To determine whether FAM3C demonstrates a similar effect on LIFR expression in human cells, a panel consisting of normal human mammary epithelial cell lines and human mammary carcinoma cell lines was sampled for comparative analysis of LIFR expression levels by immunoblotting (Fig. S1). Based on these results, two human metastatic mammary carcinoma cell lines (SKBr3 and MDA-MB-231) were selected for further study. Following shRNA knockdown of FAM3C in human cell lines, qPCR analysis revealed a measurable loss of LIFR mRNA levels relative to control cells, demonstrating a correlation between LIFR and FAM3C expression in human mammary carcinoma (Figure 2g). Immunoblot analysis of the same human cell lines showed that the loss of LIFR mRNA was sufficient to cause measurable attenuation of LIFR protein expression (Figure 2h).

### FAM3C regulates LIFR expression through STAT3

**Figure 3.**
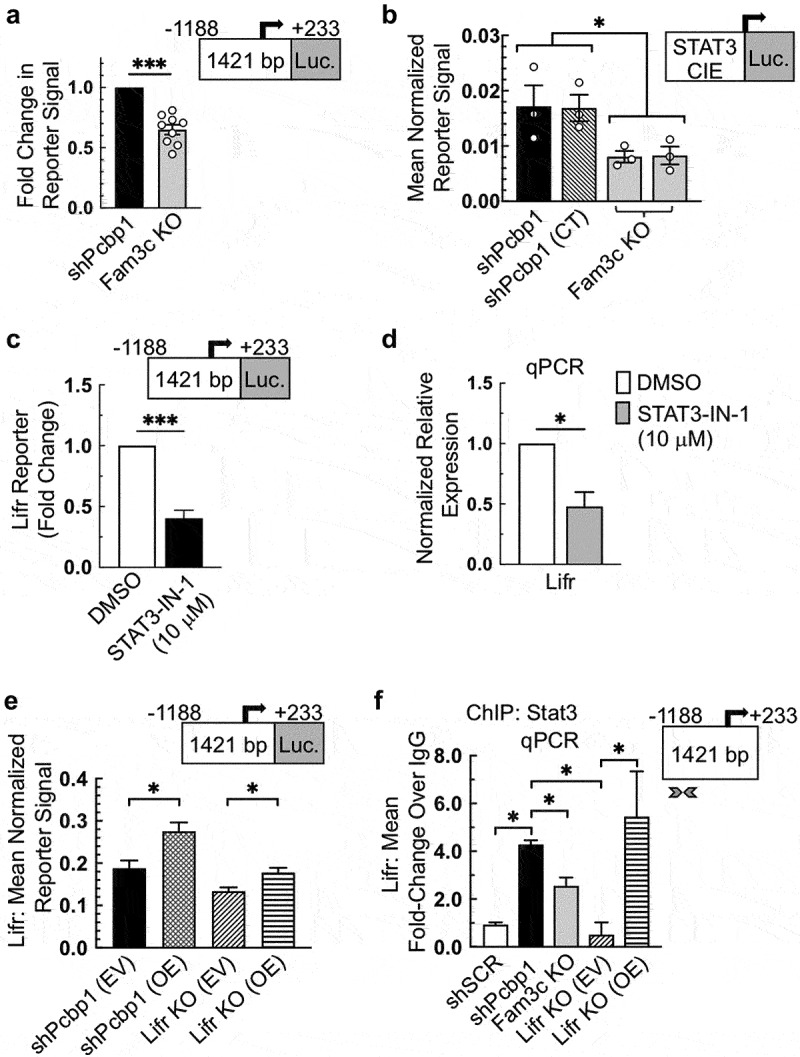
FAM3C regulates LIFR transcription through STAT3. (a) dual-luciferase assay using the indicated region of the mouse LIFR proximal promoter upstream of a firefly luciferase open reading frame (ORF), carried out in the indicated NMuMG-derivative cell lines. (b) dual-luciferase reporter assay measuring STAT3 nuclear activity, using a STAT3 cis-inducible element (CIE) upstream of a firefly luciferase ORF, carried out in the indicated NMuMG-derivative cell lines. All data points in panels “a” and “b” represent independent experiments, each performed in triplicate. Error bars represent SEM. (c) dual-luciferase assay as described in panel “a”, carried out in shPCBP1 cells treated either with DMSO (vehicle) or 10 µM inhibitor of STAT3 (STAT3-IN-1) for 24 hours. Error bars represent SEM from three independent experiments, each performed in triplicate. (d) qPCR analysis of shPCBP1 cells treated either with DMSO or 10 µM STAT3-IN-1 for 48 hours. (e) dual-luciferase assay as described in panel “a” carried out in shPCBP1 and LIFR KO cells, each transduced either with empty vector (EV) or a vector containing a viral promoter-driven mouse LIFR ORF (OE). (f) chromatin immunoprecipitation (ChIP) analysis in the indicated NMuMG derivative cell lines, following live cell cross-linking, incubation of chromatin with a STAT3 antibody, and qPCR analysis of immunoprecipitated DNA. Arrowheads indicate the region of the LIFR proximal promoter targeted by the qPCR primer set. Error bars from panels “d”, “e”, and “f” represent SEM from duplicate independent experiments, each performed in triplicate. *P < .05, **P < .01, ***P < .001 (unpaired Student’s *t*-tests). SEM, standard error of the mean.

To determine whether the loss of LIFR mRNA observed in FAM3C KO cells was due to changes in the activation of transcription at the LIFR promoter, dual-luciferase assays were performed to compare shPCBP1 cells and FAM3C KO cells following transfection of the LIFR promoter luciferase vector mentioned in the preceding section. The assay results revealed that loss of FAM3C expression significantly affected the activation of the LIFR reporter (Figure 3a). To determine whether the loss of pSTAT3 following FAM3C KO could play a role in the loss of LIFR transcriptional activation, a vector containing a STAT3 cis-inducible element (CIE) upstream of a luciferase ORF was transfected into shPCBP1 cells or FAM3C KO cells. Following transfection, dual-luciferase assays were performed, and the results showed a significant loss of the pSTAT3 reporter signal following loss of FAM3C expression (Figure 3b). To further determine whether pSTAT3 participates in the activation of LIFR transcription, shPCBP1 cells were transfected with the LIFR promoter luciferase vector in the presence of the STAT3 inhibitor STAT3-IN-1. The time of exposure and concentration of the inhibitor were based on the previous use of STAT3-IN-1 in human mammary carcinoma cell lines.^23^ The assay results showed that the pharmacological inhibition of STAT3 activation also significantly affected the activation of the LIFR reporter (Figure 3c).

To observe the effect of STAT3 inhibition on LIFR mRNA levels, qPCR was performed following treatment with STAT3-IN-1. The mRNA abundance of LIFR was significantly decreased in cells treated with the inhibitor relative to that in cells treated with vehicle only (Figure 3d). The mRNA levels of the STAT3 target genes STAT3 and MYC were also affected (Fig. S2A). An immunoblot assay was performed following treatment with STAT3-IN-1, and the results showed that the inhibitor was sufficient to cause a measurable loss of STAT3 phosphorylation (Fig. S2B). These results suggested that a feed-forward mechanism of LIFR regulation is present in NMuMG shPCBP1 cells. To determine whether increased LIFR signaling could drive LIFR gene expression, LIFR was knocked out in shPCBP1 cells using CRISPR-Cas9 (hereafter “LIFR KO cells”). A dual-luciferase assay was then performed on shPCBP1 and LIFR KO cells transduced with either EV or a vector containing a viral promoter-driven mouse LIFR ORF. The assay results revealed that LIFR reporter activity was significantly higher in cells overexpressing LIFR (Figure 3e). The relative levels of LIFR expression and pSTAT3 in the cell lines used were confirmed by immunoblotting, which demonstrated a marked increase in basal pSTAT3 levels in the cell lines with LIFR OE (Fig. S3). To determine whether STAT3 binds to DNA at the LIFR promoter locus in live cells during transcription activation, chromatin immunoprecipitation followed by qPCR (ChIP-qPCR) was performed. Briefly, shSCR, shPCBP1, and derivative cell lines were treated with formaldehyde to cross-link proteins with nuclear DNA, and chromatin extracts were immunoprecipitated using a STAT3-specific antibody. DNA isolated from the precipitates was quantified by qPCR using a primer set specific to nucleotides within the LIFR promoter sequence mentioned above. The results clearly demonstrated the binding of STAT3 to the LIFR promoter and a positive correlation between STAT3 binding activity and LIFR expression levels (Figure 3f).

### Transcriptomic analysis reveals a FAM3C/LIFR regulatory signature

**Figure 4.**
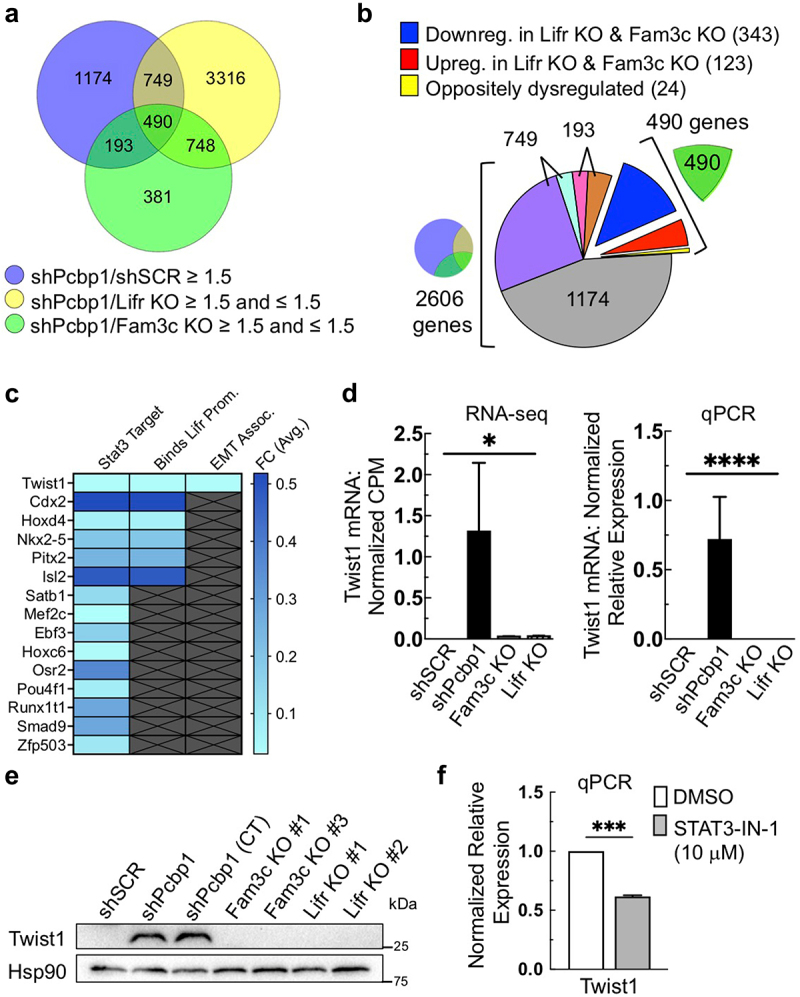
Transcriptomic analysis reveals a FAM3C/LIFR regulatory signature. (a) Venn diagram depicting the overlap of three independent RNA-Seq dataset analyses using the indicated NMuMG derivative cell lines. The center area (490) indicates the number of differentially expressed protein-coding genes (DEGs) common to all three datasets, that satisfy the indicated fold-change criteria. (b) exploded pie chart indicating the distribution of DEGs common to the overlap of datasets as indicated in the Venn diagram in panel “a”, with the indicated fractions (purple, brown, dark blue) corresponding to the downregulated DEGs following knockout (KO) of FAM3C and LIFR, relative to shPCBP1. (c) heat map indicating the average fold change in FAM3C and LIFR KO cells relative to shPCBP1 cells in the subset of 15 transcription factor (TF) genes that contain STAT3 consensus elements in their respective promoters. Center and right columns indicate if the TF gene also satisfies the indicated condition. (d) relative expression of the TWIST1 gene in the RNA-Seq data shown in panel “a”. Bars indicate average counts per million (CPM) in each group. Error bars represent SEM, *P < .05 (unpaired Student’s *t*-tests) (left); validation of TWIST1 expression by qPCR in a subset of cell lines (right). Error bars represent SEM, ****P < .0001 (two-way ANOVA). (e) Immunoblot analysis of the cell lines used for qPCR in panel “d”. (f) qPCR analysis of shPCBP1 cells treated with 10 µM inhibitor of STAT3 (STAT3-IN-1) for 48 hours. Error bars represent SEM from duplicate independent experiments, each performed in triplicate, ***P < .001 (unpaired Student’s *t*-test). Downreg., downregulated; Upreg., upregulated; prom., promoter; Assoc., associated; SEM, standard error of the mean.

Following our observation that FAM3C and LIFR regulate LIFR expression, we sought to identify additional FAM3C/LIFR-regulated genes. Transcriptomic analysis of shSCR, shPCBP1, FAM3C KO, and LIFR KO cells was performed using RNA-Seq. Briefly, unaltered shPCBP1 cells and three additional shPCBP1 CT cell lines were compared with three cell lines each of FAM3C KO and LIFR KO cells. The list of differentially expressed genes (DEGs) was aligned with a second (preexisting) RNA-Seq dataset that compared shSCR cells to shPCBP1 cells. Superimposition of the datasets identified a set of 490 DEGs that were (1) upregulated by the loss of PCBP1 relative to shSCR and (2) either upregulated or downregulated by FAM3C and LIFR KO relative to shPCBP1 cells (Figure 4a, Table S1). As expected, LIFR was found in that set of 490 DEGs. Further examination of the 490 DEGs showed that the majority were dysregulated in the same direction (either upregulated or downregulated together) following FAM3C and LIFR KO. Very few DEGs demonstrated opposite directionality relative to shPCBP1 cells (Figure 4b). This suggests that FAM3C/LIFR expression participates in maintaining the increased expression levels of a specific set of genes that are upregulated due to the loss of PCBP1. Additionally, gene ontology analysis was performed using the DAVID Knowledgebase, revealing a significant association between the shPCBP1/FAM3C/LIFR transcriptomic signature and phenotypic context involved in mammary carcinoma pathology (Fig. S4).

To examine genes in our superimposed datasets that might be indicative of a FAM3C/LIFR transcriptional cascade, 59 transcription factors (TFs) were identified within the set of 490 DEGs using the FANTOM5 database (Table S2). Using the JASPAR/TRANSFAC database, 15 of the 59 TF DEGs were identified as potential STAT3 target genes. Furthermore, the JASPAR database identified six of the 15 STAT3 target genes as potentially capable of binding to the LIFR promoter sequence used in the preceding sections. From the list of six STAT3/LIFR-participating TFs, we identified TWIST1 as a gene of interest because it was previously characterized as being involved in EMT and BCSC self-renewal (Figure 4c).^21,24–27^ Our RNA-Seq data showed that TWIST1 expression levels increased following the loss of PCBP1 expression, but then decreased following KO of either FAM3C or LIFR (Figure 4d). Changes in TWIST1 expression levels observed in the RNA-Seq data were validated by qPCR and immunoblotting (Figures. 4d,e). Additionally, qPCR results measuring TWIST1 mRNA levels in the presence of STAT3-IN-1 demonstrated a loss of TWIST1 mRNA abundance, validating our identification of TWIST1 as a STAT3-regulated gene in shPCBP1 cells (Figure 4f). The preceding analysis suggests a transcriptomic pattern downstream of FAM3C/LIFR/STAT3 modulation that is specifically implicated in mammary carcinoma pathology and that is exemplified by changes in the regulation of an established mediator of EMT and cell fate, TWIST1.

### LIFR and TWIST1 are co-regulated

**Figure 5.**
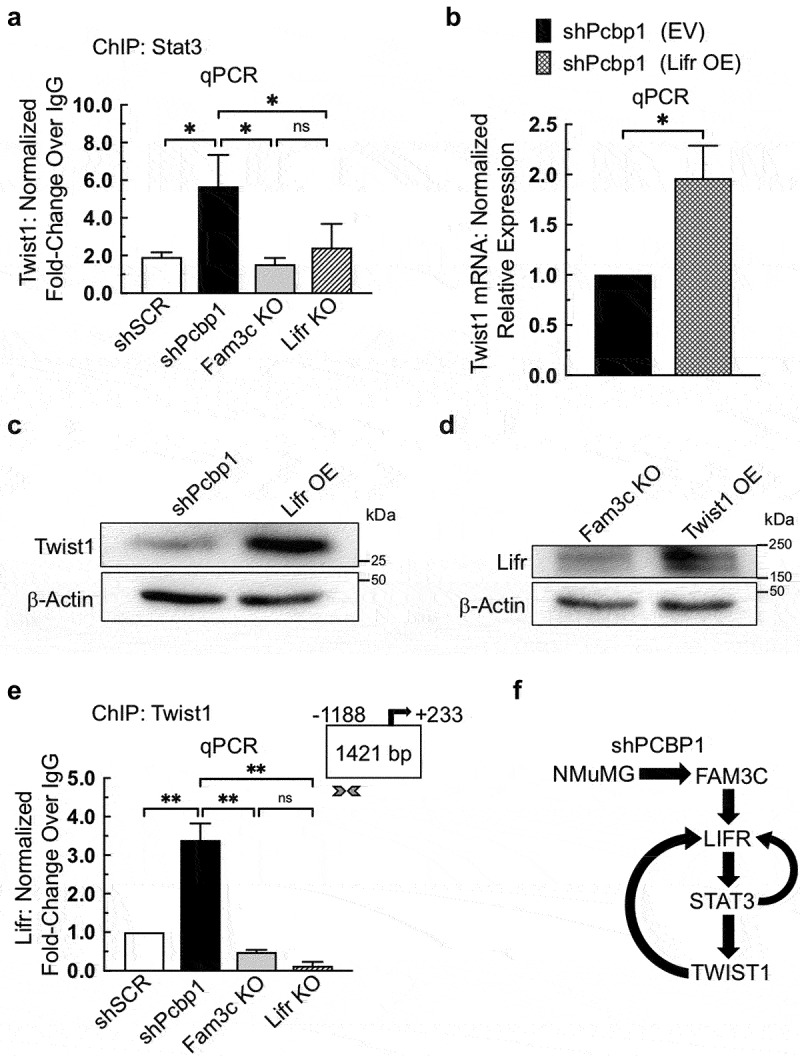
LIFR and TWIST1 are co-regulated. (a) chromatin immunoprecipitation (ChIP) analysis in the indicated NMuMG-derivative cell lines, following live cell cross-linking, incubation of chromatin with a STAT3 antibody, and qPCR analysis of immunoprecipitated DNA. Error bars represent SEM from duplicate independent experiments, *P < .05 (one-way ANOVA). (b) qPCR analysis of shPCBP1 cells transduced with either empty vector (EV) or a vector containing the mouse LIFR open-reading frame (OE). Error bars represent SEM from three independent experiments, each performed in triplicate, *P < .05 (unpaired Student’s *t*-test). (c) Immunoblot analysis of the cells shown in panel “b”. (d) Immunoblot analysis of FAM3C KO cells transduced either with EV or a vector containing the mouse TWIST1 open reading frame. (e) chromatin immunoprecipitation (ChIP) analysis in the indicated NMuMG-derivative cell lines, following live cell cross-linking, incubation of chromatin with a TWIST1 antibody, and qPCR analysis of immunoprecipitated DNA. Arrowheads indicate the region of the LIFR proximal promoter targeted by the qPCR primer set. Error bars represent SEM from duplicate independent experiments, **P<.01 (one-way ANOVA). (f) schematic diagram of the hypothesized feed-forward regulatory circuit present in shPCBP1 cells. OE, overexpression; ns, not significant; SEM, standard error of the mean.

To further validate our identification of TWIST1 as a gene regulated by LIFR/STAT3, ChIP was performed using a STAT3 antibody, as described in the preceding section, and DNA was measured by qPCR using a primer set specific to the TWIST1 promoter locus. The results demonstrated the binding of STAT3 to the TWIST1 promoter and showed that STAT3 binding activity correlates positively with TWIST1 expression in the NMuMG-derivative cell lines used (Figure 5a). To determine whether TWIST1 expression could be driven by LIFR OE, shPCBP1 cells were transduced with either EV or a vector containing the mouse LIFR ORF (LIFR OE), and qPCR was performed. The results showed a significant increase in TWIST1 mRNA abundance in LIFR OE cells relative to control cells (Figure 5b). Immunoblot analysis of the same cell lines confirmed that the increase in TWIST1 mRNA levels was sufficient to cause a measurable increase in TWIST1 protein (Figure 5c). After observing the prediction from the JASPAR database that TWIST1 could potentially bind to the LIFR promoter, we sought to determine whether TWIST1 OE could drive LIFR expression. FAM3C KO cells were transduced with either EV or a vector containing a mouse TWIST1 ORF. Immunoblot analysis revealed that the decrease in LIFR protein expression in FAM3C KO cells was partially reversed by TWIST1 OE (Figure 5d). To determine whether TWIST1 binds to DNA at the LIFR promoter in live cells, ChIP-qPCR was performed using an antibody specific to TWIST1. Using the primer set described in the preceding sections for the detection of LIFR promoter DNA, qPCR results showed binding of TWIST1 to the LIFR promoter in shPCBP1 cells. As expected, binding was not detected in shSCR, FAM3C KO, or LIFR KO cell lines, which do not express measurable levels of TWIST1 (Figure 5e). These results strongly suggest the presence of a reciprocal regulatory circuit in shPCBP1 cells that maintains LIFR expression through the feed-forward regulation of transcription. The results further suggest that shPCBP1 cells achieve increased LIFR expression and signaling through STAT3 and TWIST1 activity (Figure 5f).

### LIFR expression promotes an invasive BCSC phenotype

Previous studies by our group have characterized phenotypic changes consistent with EMT in epithelial cells with PCBP1 knockdown.^8,28,29^ Studies by our group and others have further characterized suppression of invasiveness, stemness, tumorigenesis, and metastasis by PCBP1 in various carcinomas.^5,30–33^ Additionally, numerous recent mammary carcinoma studies have implicated STAT3 signaling as a contributor to invasive potential and metastasis through its exacerbation of EMT.^34–36^ In previous studies, PCBP1 knockdown measurably altered the expression levels of epithelial and mesenchymal marker genes and dramatically changed the morphological appearance of epithelial cells to resemble that of mesenchymal cells.^5,8^ In the current study, we observed EMT morphology following knockdown of PCBP1 and further observed that LIFR OE in shPCBP1 and LIFR KO cells caused an increase in cellular elongation that often accompanies EMT (Fig. S5).

**Figure 6.**
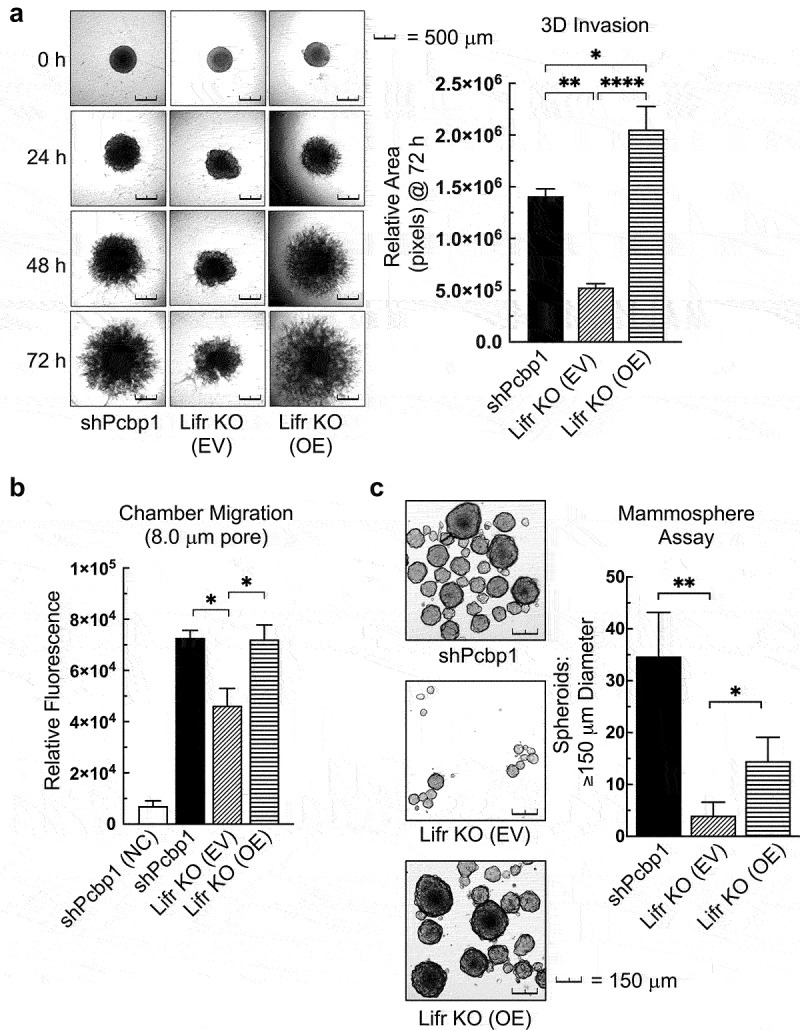
LIFR expression promotes an invasive BCSC phenotype. (a) Representative images of the indicated shPCBP1-derivative cell lines’ 3-dimensional growth in cultrex basement membrane extract at the indicated time points, taken at 5X magnification. 10% fetal bovine serum (FBS) was used as chemoattractant (left), and quantification of the mean total increase in area from zero hours (right). Error bars represent SEM from four independent experiments, *P < .05, **P < .01, ****P < .0001, (one-way ANOVA). (b) quantification of migration through polycarbonate chamber inserts (8.0 µm pore size) over 24-hours following seeding in serum-free medium. 10% FBS was used as chemoattractant. Calcein AM was used to detect only the cells that successfully migrated through the pore. Error bars represent SEM from three independent experiments, *P < .05 (unpaired Student’s *t*-tests). (c) Representative images of the indicated shPCBP1-derivative cell lines following mammosphere assay culture for 8 days, taken at 5X magnification (left). Comparison of total spheroid counts (right). Error bars represent SEM from five independent experiments, *P < .05, **P < .01 (unpaired Mann-Whitney U-Test). EV, empty vector; OE, overexpression; NC, no chemoattractant; SEM, standard error of the mean.

To determine whether LIFR expression had a direct effect on the invasiveness of shPCBP1 cells, a 3D invasion assay was performed to compare shPCBP1, LIFR KO, and LIFR KO cells with LIFR OE (LIFR KO OE cells), and relative growth patterns were quantified using a software-based method. The results showed that the loss of LIFR dramatically hinders 3D invasion, and that its OE restores invasive growth beyond the levels detected in shPCBP1 cells expressing endogenous levels of LIFR (Figure 6a). A similar result was observed when cell migration rates were measured in the same cells. LIFR KO significantly attenuated the migration rate, and LIFR OE rescued the loss of migration in LIFR KO cells. However, LIFR OE did not increase the migration rate beyond that achieved by the endogenous LIFR expression levels present in shPCBP1 cells (Figure 6b).

The mammosphere assay quantifies the capacity of mammary epithelial cells to self-renew by measuring the size and frequency of spheroid colony formation. We sought to determine whether the modulation of LIFR expression could affect the self-renewal capacity of shPCBP1 cells. shPCBP1, LIFR KO, and LIFR KO OE cells were compared by mammosphere assay, and the results showed that self-renewal was also dramatically affected by the loss of LIFR expression, and that LIFR OE partially rescued this effect (Figure 6c). When the proliferative rates were compared between the cell lines used in the preceding culture-based experiments, the differences in growth rates were not found to be significant (data not shown). These results demonstrate that LIFR expression levels dramatically affect the invasion, migration, and stemness potential of shPCBP1 cells.

### Loss of FAM3C/LIFR attenuates spheroid growth in human mammary carcinoma cells

**Figure 7.**
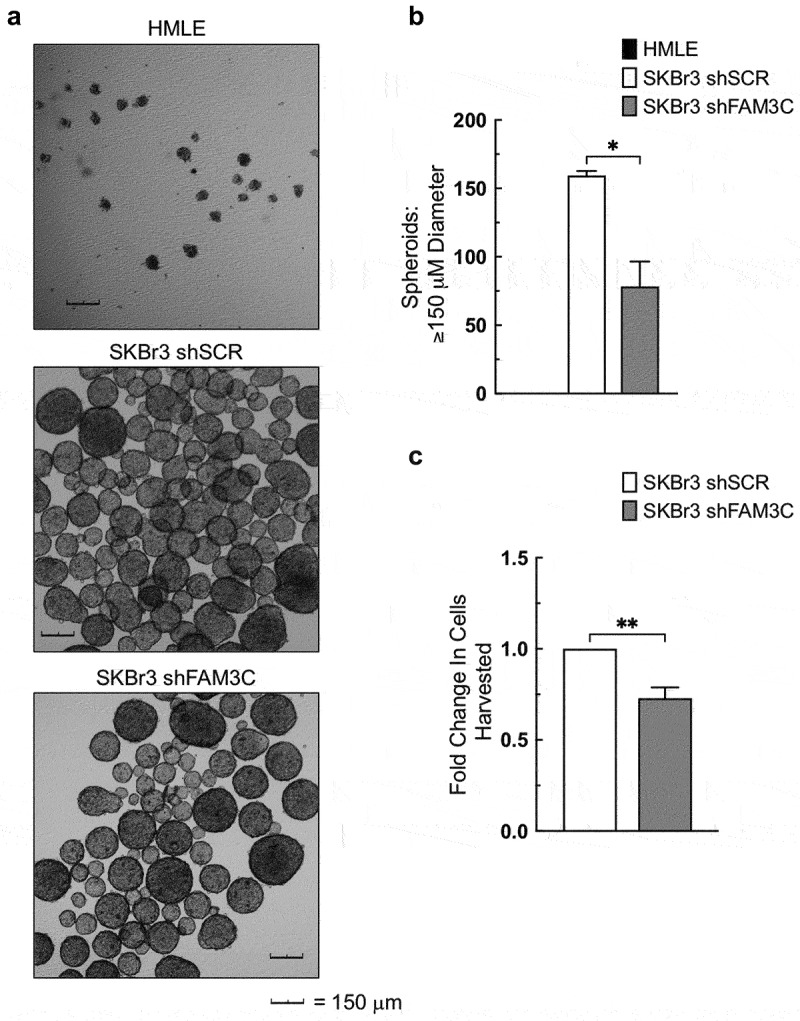
Loss of FAM3C/LIFR attenuates spheroid growth in human mammary carcinoma cells. (a) Representative images of HMLE cells and the indicated SKBr3-derivative cell lines following mammosphere assay culture for 8 days, taken at 5X magnification. (b) Comparison of total spheroid counts from the cells shown in panel “a”. (c) Comparison of total cell counts from the SKBr3 cells shown in panel “a”, following trypsinization and cell counts of harvested spheroids. Error bars represent SEM from duplicate independent experiments, *P < .05, **P < .01 (unpaired Student’s *t*-tests). SEM, standard error of the mean.

Our group previously observed attenuation of spheroid growth by mammosphere assay in shPCBP1 cells following knockdown of either FAM3C or LIFR.^7^ To determine whether loss of FAM3C/LIFR expression would similarly affect spheroid growth in human cells, SKBr3 cells with shRNA-mediated knockdown of FAM3C (shFAM3C cells) were compared with control cells (HMLE normal human mammary epithelial cells and SKBr3 shSCR cells) by mammosphere assay. SKBr3 shFAM3C cells showed a measurable reduction in spheroid formation compared to SKBr3 shSCR cells, and, as predicted, normal HMLE cells were not viable under these growth conditions (Figures. 7a,b). Notably, the size of the spheroids in both groups was variable, with large spheroids being present in both groups. To determine whether the proliferative rate of the cells under these conditions was also measurably attenuated by FAM3C knockdown, harvested spheroids were trypsinized, and pooled cells were counted following each experiment. Although control and shFAM3C cells were seeded in equal numbers, control cells showed a greater increase in growth after eight days (Figure 7c). These results suggest that the loss of FAM3C/LIFR expression attenuates both the self-renewal capacity and viability of human mammary carcinoma cells under non-adherent, serum-free growth conditions *in vitro*.

## Discussion

PCBP1 is a multifunctional nucleic acid-binding protein that has been implicated in the pathogenesis of various carcinomas through its suppression of EMT and tumorigenesis.^4^ Although a variety of PCBP1-dependent mechanisms have been described, those responsible for disease progression in mammary carcinomas remain largely unknown. In the current study, we defined a mechanism of phenotypic change in murine mammary epithelial cells lacking PCBP1 function. Furthermore, we demonstrated aberrant expression of the LIFR gene following the loss of PCBP1 and illustrated a mechanism of LIFR maintenance in mouse and human tumorigenic mammary epithelial cells. We also provided data that describe a signature of gene regulation downstream of the FAM3C/LIFR interaction, and that identified TWIST1 as a transcription factor that participates in the maintenance of LIFR expression. Finally, we demonstrated that the aberrant expression of LIFR in shPCBP1 cells supports increased invasion, migration, and self-renewal capacity.

Our previous study demonstrated the effect of FAM3C or LIFR knockdown in shPCBP1 cells by revealing that increased self-renewal *in vitro* and decreased tumor burden and metastases *in vivo* were dependent on FAM3C-induced LIFR-dependent pSTAT3.^7^ Preclinical studies by other groups have likewise demonstrated that LIFR expression promotes breast cancer progression. For example, Zeng et al. demonstrated that upregulation of LIFR occurs following histone deacetylase inhibition (HDACi) and subsequently causes increased expression of STAT3-target genes associated with resistance to apoptosis. Zeng et al. concluded that LIFR-induced pSTAT3 promotes disease by decreasing the efficacy of HDACi therapies.^37^ More than ten additional examples of preclinical characterization of LIFR/JAK/STAT3 in nine additional cancer types can be associated with the conclusion that increased LIFR expression promotes disease progression.^38^ However, several studies have shown that increased LIFR expression can suppress disease progression through its induction of JAK/STAT3 and other signaling pathways, suggesting that the role of LIFR is “pleiotropic” due to its range of potential downstream regulatory effects. For example, Johnson et al. suggested that although LIFR expression increases following HDACi treatment, upregulation of STAT3-target genes not only induces stemness, but also dormancy in cancer cells.^14^ Although Johnson et al. further characterized the induction of LIFR expression following HDACi treatment in a later study, and reached some conclusions that were similar to those of Zeng et al., it was demonstrated that LIFR upregulation following HDACi treatment drives cancer cell dormancy, potentially prolonging patient survival.^39^ Several additional studies by other groups have provided preclinical *in vivo* evidence suggesting that LIFR suppresses disease progression by inducing JAK/STAT3 and other pathways, including HIPPO and PI3K/AKT.^40,41^ Although examination of the preponderance of pan-cancer preclinical data characterizes LIFR as a promoter of carcinoma pathology (approximately two-thirds of examples), a considerable amount of data has shown that LIFR can act as a suppressor of disease progression, prompting the need for further research to delineate the breadth of LIFR-dependent intracellular mechanisms in neoplastic tissues and the extent to which the tissue of origin may act as a determinant.^38^

In the current study, we sought to build upon our previous findings by identifying key intracellular mechanisms and dysregulated genes downstream of FAM3C/LIFR interaction. Our identification of a feed-forward mechanism of LIFR regulation aligns with a previous investigation that demonstrated an increase in LIFR mRNA levels following leukemia inhibitory factor-induced pSTAT3.^42^ Additionally, examination of the genome-wide occupancy of STAT3 by high-throughput sequencing (ChIP-seq) in MDA-MB-231 TNBC cells revealed an example of STAT3 binding to the 5’ region near the promoter of the LIFR gene.^43^ Additional ChIP-seq datasets from murine embryonic fibroblasts and oligodendrocyte progenitors have revealed that STAT3 binds to the LIFR transcription start site region.^44,45^ Furthermore, our identification of LIFR expression/pSTAT3 signaling as promoters of invasion, migration, and self-renewal aligns with previous findings in multiple carcinoma types, including breast cancer.^46–52^ Finally, the loss of self-renewal and viability in human mammary carcinoma cells (SKBr3) following FAM3C knockdown suggests that FAM3C-mediated maintenance of LIFR expression may be required to maintain the BCSC phenotype in human breast cancer. However, further studies using animal models are required to determine how LIFR expression directly affects the metastatic potential of the invasive mammary carcinoma cell lines used in the current study.

It is important to note that our experimental context showcased the manipulation of LIFR expression in tumorigenic cells that have previously undergone EMT. The role of EMT in the promotion of disease progression and the degree to which it affects the phenotype of cancer cells can potentially vary and may explain some of the differences in the conclusions drawn by other groups attempting to define the role of LIFR in disease progression. Our previous studies have clearly demonstrated the role of shPCBP1-mediated EMT in the progression of mammary carcinoma *in vivo* and was therefore selected as an appropriate experimental context for further characterization of FAM3C/LIFR/STAT3 participation in the regulation of pathological features.

Previous studies have identified TWIST1 as a transcription factor that plays a role in regulating the genes responsible for EMT and stem cell self-renewal.^21,24,26,53^. Zhao et al. demonstrated STAT3-dependent TWIST1 induction and subsequent STAT3/TWIST1-dependent EMT in the lung-metastatic derivative (LM2–4175) MDA-MB-231 human mammary carcinoma cell line.^21^ Similar evidence was presented by Lin et al. using A549 lung adenocarcinoma cells and by Cho et al. using PC-3 human prostate cancer cells.^54^ Surprisingly, TWIST1 overexpression did not significantly increase invasion, migration, or self-renewal of shPCBP1 cells in the current study, nor did it rescue the loss of these properties in LIFR KO cells (data not shown). The direct influence of TWIST1 on EMT, stemness, and migration/invasion in our model requires further investigation. However, we were able to determine that TWIST1 participates in the regulation of LIFR; therefore, it is plausible that TWIST1 influences the phenotype of shPCBP1 cells through the propagation of LIFR-dependent signaling. Our transcriptomic data also identified TWIST2 as a dysregulated transcription factor that might be involved in driving phenotypic changes in shPCBP1 cells. Although the expression of TWIST2 was significantly increased in shPCBP1 cells relative to shSCR cells, preliminary experiments failed to reveal how a modest downregulation of TWIST2 following FAM3C or LIFR KO impacts the shPCBP1 phenotype (data not shown).

Our transcriptomic analysis examined the overlap of DEGs that were upregulated by the loss of PCBP1 and dysregulated (either up- or down-regulated) by the loss of FAM3C and LIFR expression. Seventy percent of the DEGs identified were downregulated by FAM3C and LIFR KO, which allowed us to assert that the upregulation of FAM3C and LIFR following the loss of PCBP1 provides an interaction that allows cells to maintain the expression of a specific set of genes. We focused on the genes downstream of pSTAT3, but it is important to note that LIFR has been shown to activate signal transduction through several additional pathways, including MAPK, PI3K/AKT/mTOR, and HIPPO-YAP, in a manner that is selectively responsive to coupling with other members of the IL-6 family of cytokine receptors.^15,41,55–57^ Additionally, there are several examples of carcinoma studies that implicate LIFR/STAT3 in regulatory “cross-talk” with additional signaling pathways including HEDGEHOG and WNT.^58–60^ Preliminary pathway analysis of our transcriptomic data revealed associations between our set of 490 DEGs and both the HEDGEHOG and WNT pathways; however, delineation of the relevant mechanisms related to this study will require further analysis (data not shown).

Ongoing analysis of our transcriptomic data may reveal additional DEGs required for invasion and migration, which are regulated by LIFR expression and signaling. We identified the matrix-metalloprotease 2 (MMP2) gene as a candidate DEG in our dataset, and further characterization of its expression in shPCBP1 cells may enable connection to FAM3C/LIFR/STAT3 signaling. MMP2 has been shown to be directly regulated by STAT3, and examination of ChIP-seq data from MDA-MB-231 cells showed that STAT3 binds to the 5’ region near the MMP2 promoter.^43,61,62^ Additionally, recent data have characterized the role of MMP2 in invasive breast cancer and metastatic melanoma and demonstrated a correlation between STAT3 activity and MMP2 expression.^61,63,64^ Coincidentally, Schmidt et al. identified a loss of MMP2 expression following knockdown of FAM3C in human breast cancer cells.^65^

In light of the fact that an estimated 90% of breast cancer-related deaths are caused by metastatic disease, it is appropriate to continue research to identify how transformed cells gain the ability to invade adjacent tissues and evade sensitivity to chemotherapy.^66^ Here, we describe a signaling mechanism in a model system that exemplifies the relevant characteristics of advanced mammary carcinoma pathology, and we provide evidence of parallel phenomena in human breast cancer cells. Although further studies are needed to formulate interventions for the pathology that results from loss of PCBP1 function, we believe that our findings elucidate a key component of PCBP1’s role in suppression of the metastatic cascade.

## Experimental procedures

### Cell culture

NMuMG, HEK293T, HMLE, MCF10A, MCF7, SKBr3, and MDA-MB-231 cells (ATCC) were maintained at 37°C and 5% (v/v) CO_2_ in a humidified incubator. MCF7 cells were generously gifted by Dr. Wenjian Gan (MUSC). NMuMG, HEK293T, MCF7, and MDA-MB-231 cells were cultured in DMEM (Corning) 4.5 g/L glucose supplemented with 10% fetal bovine serum (FBS) (Atlanta Biologicals). SKBr3 cells were cultured in McCoy’s 5A Modified Medium (Thermo Fisher Scientific) supplemented with 10% FBS. HMLE cells were cultured in DMEM-F12 (Gibco) supplemented with 5% calf serum (VWR), 0.5 μg/ml hydrocortisone, 10 µg/ml insulin, and 20 ng/ml epidermal growth factor (Corning). MCF10A cells were cultured in DMEM F-12 supplemented with 5% horse serum, 20 ng/ml epidermal growth factor, 100 ng/mL cholera toxin (Sigma), 10 µg/mL insulin (Sigma), and 0.5 µg/mL hydrocortisone (Sigma). All cell lines were supplemented with 1% antibiotic/antimycotic (Gibco) and 0.02% prophylactic plasmocin (InvivoGen).

### CRISPR-Cas9

Single-guide RNA (sgRNA), tracrRNA, and Cas9 protein (IDT) were assembled into a complex according to the manufacturer’s specifications (Lonza) and electroporated into cells using Amaxa Nucleofector II (Lonza). sgRNA pairs were selected for excision of a ~ 125 bp DNA fragment in the coding sequence of the target gene. The electroporated cells were serially diluted in 96-well culture dishes to isolate KO candidates from discrete colonies. Candidate cell lines were screened by PCR using primers flanking or nested within the excision. Cell lines with positive PCR results were further screened by immunoblotting to confirm KO of the target gene. The sgRNA and PCR primer sequences are listed in Table S3.

### Lentiviral transduction

Lentivirus was produced for transduction of either shRNA or protein overexpression vectors using the 2^nd^ generation system. An envelope plasmid (pMD2.G), packaging plasmid (psPAX2), and either an shRNA-containing vector (pLKO.1-puro, Addgene #8453) or mammalian protein expression vector (pLenti-CMVie-IRES-BlastR, Addgene #119863) were transfected into HEK293T cells using Lipofectamine 3000 (Thermo Fisher) in Opti-MEM serum-free medium (Gibco). Virus-containing supernatant media (5 mL) were collected after 36–48 hours, strained through a .40 µm filter, diluted in DMEM by a factor of 5–10, and added to target cells with 8 µg/mL polybrene. Following incubation for 24–48 hours, 1 µg/mL puromycin or 10 µg/mL blasticidin was added to the culture medium to select the target cells. The shRNA sequences for human shSCR and for FAM3C knockdown are listed in Table S3.

### Immunoblotting

Whole cell lysates were produced by rinsing cells grown on culture plates twice with PBS, prior to scraping the cells into a small volume of PBS. Cells were pelleted by centrifugation at 300 × G in microcentrifuge tubes, and an appropriate amount of RIPA lysis buffer (50 mM Tris-Cl, pH 7.6, 1% NP-40, 12 mM sodium deoxycholate, 0.1% sodium dodecyl sulfate (SDS), 165 mM sodium chloride) was added. The cells were kept on ice with occasional mixing for ~45 min and then centrifuged at 16,000 × G at 4°C for ten minutes. The supernatants were then transferred into fresh tubes and stored at −20°C. Protein concentrations were measured using a Pierce Micro BCA Protein Assay Kit (Thermo Fisher Scientific). Samples were denatured by adding an appropriate volume of Laemmli sample buffer (62.5 mM Tris-Cl pH 6.8, 1% SDS, 10% glycerol, 50 mM dithiothreitol (DTT), 0.001% bromophenol blue), followed by incubation at 95°C for 5 min. Denatured samples were resolved by electrophoresis through polyacrylamide gels ranging from 7.5% to 12.5% and electrotransferred onto polyvinylidene fluoride (PVDF) membranes at a constant current for 16–20 hours. Membranes were incubated in blocking buffer (5% skim milk in Tris-buffered saline with 0.01% Tween-20, TBST) for one hour at room temperature (RT), and then incubated for 2–24 hours at 4°C in blocking buffer with the addition of a primary antibody to detect the protein of interest. The following primary antibodies were used: LIFR (Santa Cruz #sc -515,337), FAM3C (Sigma #AV44904), pSTAT3 (Cell Signaling #9145), STAT3 (Cell Signaling #9139), GAPDH (Santa Cruz #sc -32,233), β-Actin (Santa Cruz #sc -47,778), TWIST1 (Cell Signaling #90445), and HSP90 (Santa Cruz #sc -13,119). After primary antibody incubation, the membranes were rinsed 3 X 10–15 minutes in TBST and then incubated in blocking buffer with the addition of the appropriate secondary antibody for 0.5–1.5 hours at RT. The following horseradish peroxidase (HRP)-conjugated secondary antibodies were used: goat anti-mouse IgG (Thermo Fisher #31430; 1:10,000) and goat anti-rabbit IgG (Thermo Fisher #31460; 1:10,000). After incubation with the secondary antibody, the membranes were washed again as described above. Bands were detected by adding 1–2 mL of HRP substrate (EMD Millipore) directly onto the membrane. Images were acquired using ChemiDoc MP (Bio-Rad) and processed using Image Lab software (Bio-Rad).

### Quantitative real-time PCR

RNA was extracted from cells using TRIzol reagent (Ambion), according to the manufacturer’s protocol. RNA concentrations were determined using a Nanodrop 2000 spectrophotometer (Thermo Fisher Scientific). cDNA was synthesized using QScript cDNA Supermix (Quantabio) according to the manufacturer’s protocol for a final concentration of 100 ng/mL. PCR reactions were carried out in 10 µL volumes using 2X iTaq Universal SYBR Green Supermix (Bio-Rad), with a final cDNA concentration ranging from 500 pg to 6 ng/µL. Primers were used at a final concentration of 570 nM. A list of the primer sequences used for target genes and for internal control “housekeeping genes” can be found in Table S3. Nucleic acid standards for the calculation of PCR efficiency were formulated using the purified PCR amplicons. An eight-fold dilution series of four standards ranging from concentrations of approximately 73 to 300 pg/µL was used to calculate a standard curve for each primer set used. The negative controls consisted of cDNA samples prepared without the QScript Supermix. PCR reactions were carried out in triplicate in 384-well opaque, white-skirted plates on a C1000 Touch Thermal Cycler (Bio-Rad) coupled to a CFX384 Real-Time System (Bio-Rad). Data were analyzed using CFX Maestro software (Bio-Rad), and normalized relative expression was calculated using the Eff^(-ΔΔCT)^ method, where Eff = 2 × (PCR efficiency% ÷ 100), CT = thermal cycle threshold of detection, ΔCT = (target gene CT – internal control gene CT), and ΔΔCT = (experimental sample ΔCT – control sample ΔCT).

### Dual-luciferase assay

A DNA sequence corresponding to the mouse LIFR regulatory region (Table S3) was amplified from genomic DNA extracts and ligated into the pGL3-Basic vector (Promega). Approximately 100 K cells in 12-well culture plates were co-transfected with ~1 µg pGL3 and ~ 500 ng pNL3.1 (Promega) and allowed to grow for 24 h. The cells were then trypsinized and resuspended in DMEM containing 10% serum, centrifuged at 300 × G and washed 2X with PBS with repeated centrifugation. Cells were then resuspended in 200 µL of PBS and distributed into 96-well opaque white assay plates (triplicate wells at 60 µL). Luminescence was measured using the Nano-Glo Dual-Luciferase Reporter Assay System (Promega) according to the manufacturer’s protocol using a Molecular Devices Spectramax iD5 Multi-Mode Microplate Reader. The relative normalized reporter signal was calculated by first subtracting “background signal” firefly luminescence (pGL3) acquired from pGL3-Basic “empty vector” control transfections from each experimental transfection, then dividing the remaining firefly luminescence values by the Nano-Luc luminescence (pNL3.1) values generated by the same well to determine the normalized reporter signal per well. Finally, the mean normalized reporter signal was determined from triplicate wells and compared between experimental groups. For experiments using the STAT3 CIE reporter, the pGL3 vector was replaced with the pGL4.47 vector (Promega). For experiments using STAT3 inhibition, the cells were seeded in DMEM and allowed to grow overnight. Immediately prior to transfection, the culture medium was replaced with a medium containing STAT3-IN-1 (10 µM final concentration) or an equal volume of DMSO.

### Mammosphere assay

Culture plates containing adherent cells were trypsinized using trypsin-EDTA (Gibco), resuspended in DMEM containing 10% FBS, and centrifuged at 300 × G. The medium was aspirated, and the cells were resuspended in serum-free DMEM to a concentration of ~ 500 X10^3^ cells per mL. Cells were then counted manually using trypan blue under 10X magnification, using disposable cell counting slides (KOVA Glasstic #87144). Cells were diluted to a concentration of 100 X10^3^ cells per mL and strained through a 40 µm mesh strainer into 5 mL of mammosphere medium (final concentration 2.0 X10^3^ cells per mL for mouse cells, and 6.7 X 10^3^ cells per mL for human cells). The mammosphere medium consisted of DMEM F12 (Gibco) supplemented with recombinant human epidermal growth factor (20 ng/mL), recombinant human basic fibroblast growth factor (20 ng/mL), B-27 supplement (Thermo Fisher #17504044), and 1% antibiotic/antimycotic (Gibco). Strained cells were distributed into six wells of a 24-well ultra-low attachment culture plate (Corning #3473) at a volume of 750 µL per well (1.5 X10^3^ cells per well for mouse cells, and 5.0 X 10^3^ cells per well for human cells). Cells were allowed to grow undisturbed for 8 days, then imaged and counted manually using a 150 µm cutoff threshold. After 8 days of growth, human mammospheres were collected and pooled in 15 mL conical tubes, and PBS was used to rinse wells to collect spheroids. Trypsin-EDTA (Gibco) was added directly to spheroid suspensions in 15 mL tubes (final concentration ~ 0.1%), and incubated at 37°C for 10 minutes, with occasional inversion of the tube. Tubes were then centrifuged at 300 X G for 5 minutes to pellet cells, followed by resuspension in 500 µL of DMEM and manual counting as described above.

### 3D invasion assay

Cells were trypsinized using trypsin-EDTA (Gibco), resuspended in DMEM containing 10% FBS, counted manually as described above, and then diluted to 1.0 X10^6^ cells/mL concentration in mammosphere medium (see above for recipe). The cells were again diluted to 1.0 X10^5^ cells/mL in 1 mL of mammosphere medium, and 50 μ L (5000 cells) of each cell sample was added (in duplicate) to a 96-well ultra-low binding U-shaped bottom culture plate (Corning #07-202-463). Cells were then centrifuged at 300 × G for 5 min and allowed to grow for 24 h at 37°C to form spheroids. Following visual confirmation of spheroid formation, culture plates were chilled on ice for 15 minutes, and 37.5 μ L of ice cold Cultrex Basement Membrane Extract (R&D Systems #3432-010-01) was added to each well and centrifuged at 300 × G for 5 minutes. Cells were then incubated at 37°C for 1–2 hours and initial time-point images were taken. DMEM lacking serum (control wells) or containing 10% FBS (chemoattracted wells) was then overlaid (150 μ L) onto each well. Spheroids were imaged again at 24-hour intervals thereafter and incubated for 72 additional hours. The relative increase in area was calculated by measuring the pixel area at the 72-hour time point and then subtracting the pixel area from the initial time point. Pixel area was calculated using ImageJ software (version 2.9.0/1.53t) equipped with the Izrs1.zip installation of “Analyze_Spheroid_Cell_Invasion_In_3D_Matrix” tool (Volker Baecker, 2017).

### Imaging of mammosphere and 3D-invasion assays

Mammospheres and 3D-invasion spheroids were imaged using a Leica (DM IL LED Fluo) phase-contrast light microscope at 5X magnification, equipped with a USB 2.0 digital camera (AmScope MU 500, 5.1 MP) attached to a PC (Dell Optiplex 380) running a Windows 7 operating system. The mammosphere diameter was measured using AmScope software (version X86 4.11) calibrated with an improved Neubauer hemocytometer grid.

### Chromatin immunoprecipitation/DNA isolation

Cells were grown in 150 mm culture plates until 80–90% confluent and were cross-linked, followed by chromatin isolation and analysis using the SimpleChIP^Ⓡ^ Enzymatic Chromatin IP Kit (Cell Signaling, #9003) according to the manufacturer’s specifications. 8–10 μ g of chromatin was then incubated for 24 h at 4°C with antibodies against STAT3 (Cell Signaling #9139, 0.275 µg/IP), TWIST1 (Santa Cruz #sc -81,417, 3.5 μ g/IP), or an equal amount of nonspecific mouse IgG (Cell Signaling #5415S). Antibodies were collected using magnetic beads and DNA was purified according to the manufacturer’s specifications. qPCR was then carried out as described above using primer sets specific to the genomic region of interest (Table S3). The relative fold-change of DNA abundance over IgG was calculated using the Eff^(-ΔΔCT)^ method, where Eff = 2 × (PCR efficiency% ÷ 100), CT = the thermal cycle threshold of detection, ΔCT = (target gene IP CT – target gene input CT), and ΔΔCT = (target gene IP ΔCT – IgG IP ΔCT). 2% of each chromatin sample was set aside to be used as an input sample, and the CT values of input samples were reduced by a value 5.64 (2^5.64^ = 50) to adjust for the 1:50 dilution factor relative to the IP sample.

### Chamber insert migration assay

Cells were trypsinized using trypsin-EDTA (Gibco), resuspended in phenol red-free DMEM containing 10% FBS, counted, and then diluted in serum-free phenol red-free DMEM at a concentration of 200 × 10^3^ cells/mL. The cell suspension (200 mL, 4 × 10^4^ cells) was then added to the top chamber of a 12-well polycarbonate membrane-containing insert with an 8.0 µm pore size (CellTreat #230633). Next, 800 µL of either serum-free DMEM (control inserts) or DMEM containing 10% FBS was added to the bottom chamber and the cells were incubated at 37°C for 24-hours. The media were then aspirated from the bottom chambers, 200 µL of 0.05% trypsin-EDTA containing 2.5 µg/mL calcein AM (Sigma) was added to the bottom chambers, and inserts were incubated for 30 min at 37°C to allow migrated cells to detach from the underside of the insert. The plates were gently tapped to aid detachment of the cells, and the inserts were then removed. The 200 µL volume was then mixed gently, and 180 µL was added to an opaque white 96-well assay plate (3 × 60 µL/well). Fluorescence was measured using a Molecular Devices Spectramax iD5 Multi-Mode Microplate Reader with excitation and emission wavelengths of 488 and 520 nm, respectively. The relative fluorescence was quantified by generating a standard curve using serial dilution of a known quantity of calcein AM-treated cells. Background subtraction was performed by measuring a well containing a 0.05% trypsin-EDTA/calcein AM solution without cells.

### RNA-Seq

Cells were seeded on 60 mm culture plates and grown until ~ 75% confluence, trypsinized, washed twice with PBS, pelleted, and placed on ice. Total RNA was extracted from cells using the simplyRNA Cells kit (cat # AS1390) with Maxwell RSC 16 (Promega) according to the manufacturer’s specifications. RNA QC, along with that of the downstream libraries (below), was performed using a 4200 TapeStation (Agilent). One mg of total RNA was used to construct libraries with the New England Biolabs NEBNext^Ⓡ^ rRNA Depletion Kit (Cat# E6310X) and Ultra II Directional RNA Library Prep Kit for Illumina (Cat# 7760 L), according to the manufacturer’s instructions. Dual-indexed libraries were pooled and sequenced at VANTAGE (Vanderbilt University Medical Center) on an Illumina NovaSeq 6000 (S4 flow cell) to a depth of approximately 50 million paired-end 150 bp reads per library. Files containing paired-end reads (in.fastq.gz format) were uploaded to the Partek Flow web-based software platform (version 10.0.21). Reads were trimmed and aligned to the GRCm38 (mm10) mouse genome assembly using the Bowtie2 applet. Aligned reads were annotated, and differential expression analysis was performed using the DESeq2 applet. See the “Data Availability” subsection for access to raw and processed data files.

### STAT3 inhibition

Cells were treated with DMEM supplemented with either 5 or 10 µM STAT3-IN-1 (MedChemExpress) in DMSO, or DMSO only, for 24 or 48 h, as described previously. Cell seeding was adjusted as necessary to compensate for the slightly diminished proliferative rate in the 10 µM STAT3-IN-1 treatments. Cells were seeded to permit continuous growth over the indicated time periods, without the need for media changes.

### Gene ontology analysis

The list of gene IDs (official gene symbols) derived from transcriptomic data (Table S1, S2) was fed directly into the analysis wizard of the DAVID Bioinformatics resource.^67,68^ The functional annotation tool and gene ontology were selected. The data returned for biological process annotations were used to generate the graphic visualizations shown in the supplemental data section of this article.

### Statistical methods

The experimental results were analyzed using GraphPad Prism (version 9.5.1). Bars in the bar graphs represent the experimental mean, and error bars represent the standard error of the mean, unless otherwise indicated. Independent unpaired Student’s *t*-tests were used for individual comparisons, and one-way or two-way ANOVA was used for group comparisons, where applicable. Transcriptomic data analysis for individual genes was performed by DESeq FDR step up, which included a post-hoc test (q-value) for multiple comparisons q < 0.07 was considered significant. Superimposition of RNA-Seq datasets generated DEG lists based on fold-change and were agnostic to q-values. Individual candidate DEGs expression levels were validated by qPCR analysis where applicable. Post-hoc tests were not performed unless otherwise indicated. All experiments were repeated at least twice, and statistical significance was set at *P* < .05. P-values for gene ontology data were generated using methods within the DAVID Bioinformatics Database.

## Supplementary Material

Supplemental Material

## Data Availability

The RNA-Seq data generated in this study are publicly available in the Gene Expression Omnibus (GEO) under GSE234882.
