# Supplementary figures and images for: PCBP1 regulates LIFR through FAM3C to maintain breast cancer stem cell self-renewal and invasiveness
Source: Cancer Biol Ther. 2023 Nov 6;24(1):2271638. doi: 10.1080/15384047.2023.2271638 (PMC10629429; doi:10.1080/15384047.2023.2271638)

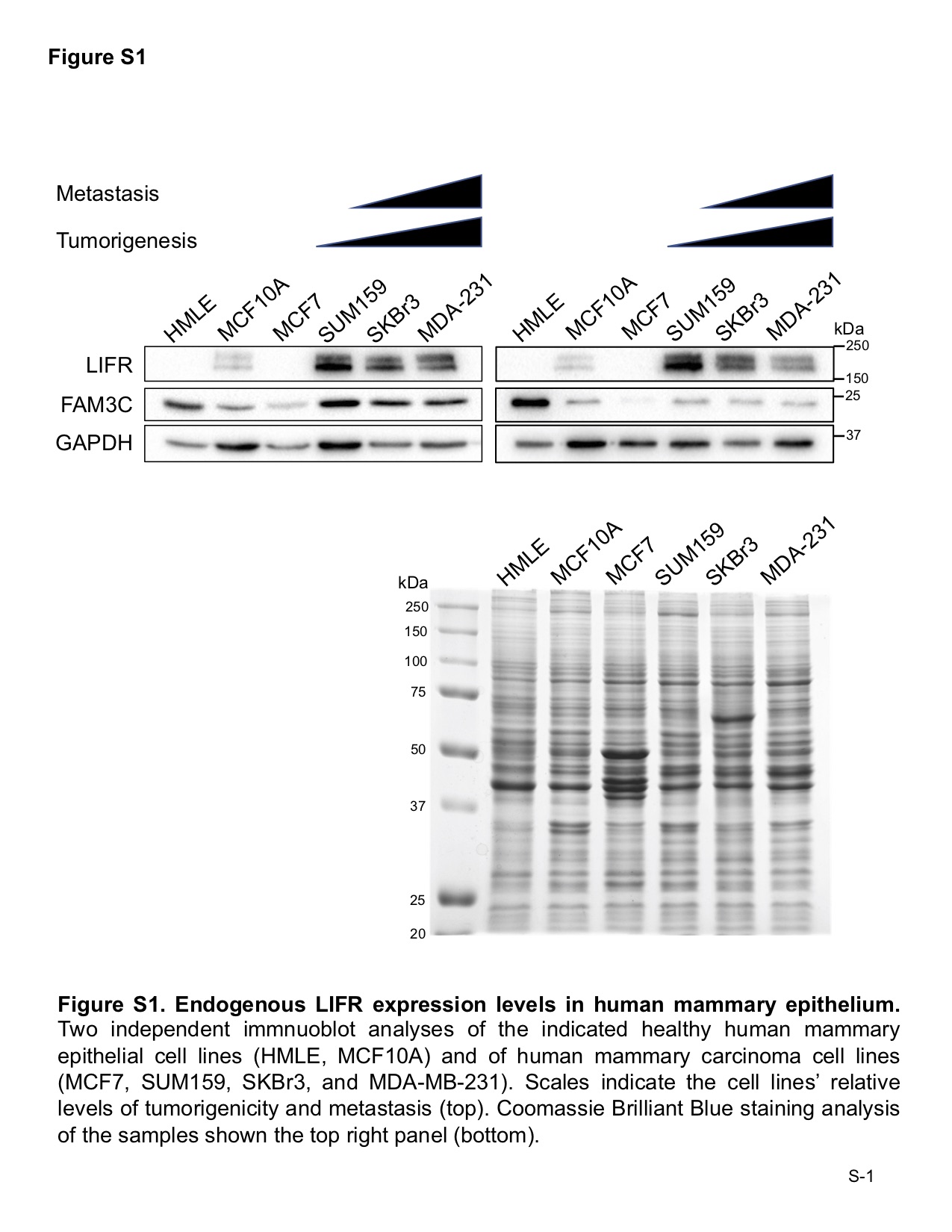

Supplement: Supplemental Material [file KCBT_A_2271638_SM4140.zip › Streitfeld_Fig S1_REVISED.jpg]

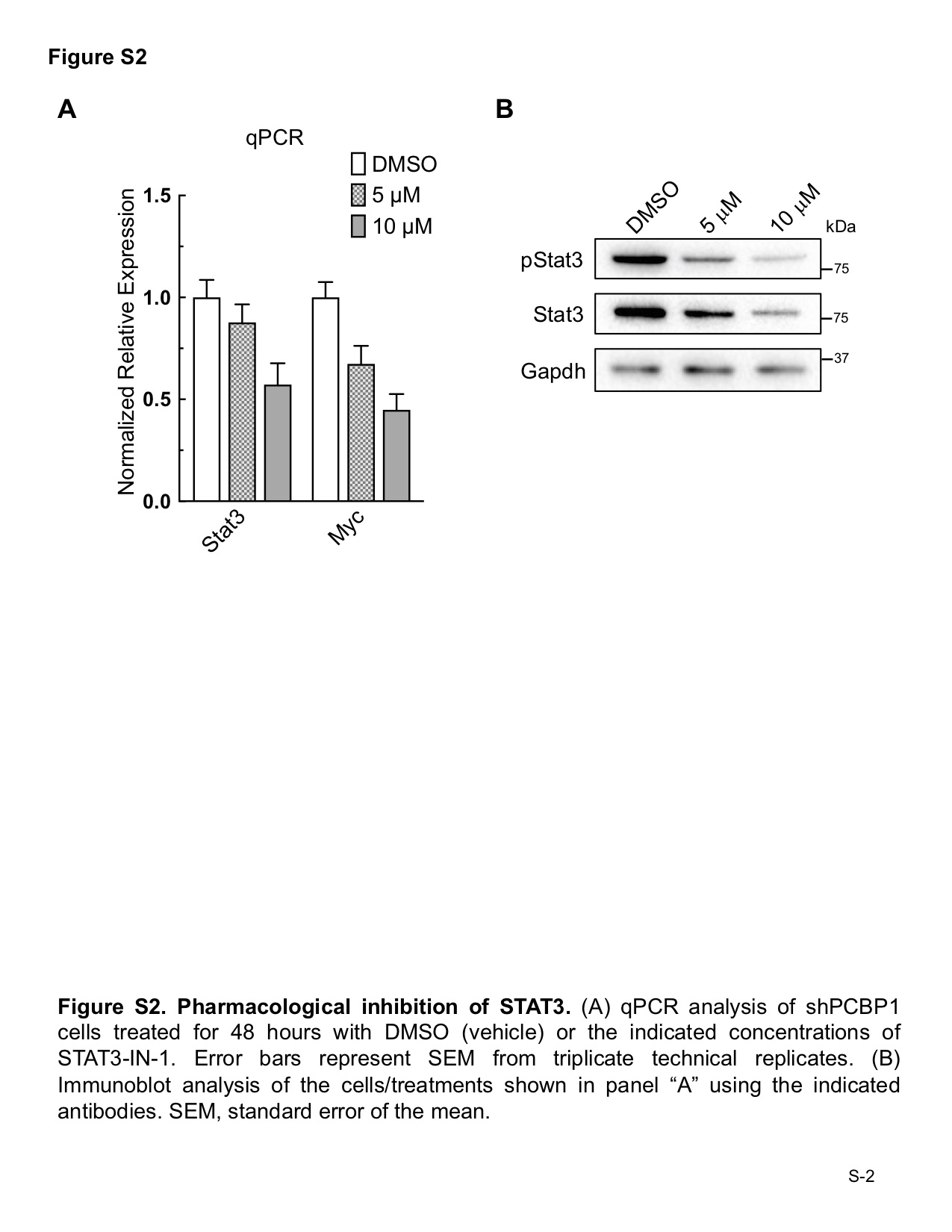

Supplement: Supplemental Material [file KCBT_A_2271638_SM4140.zip › Streitfeld_Fig S2_REVISED.jpg]

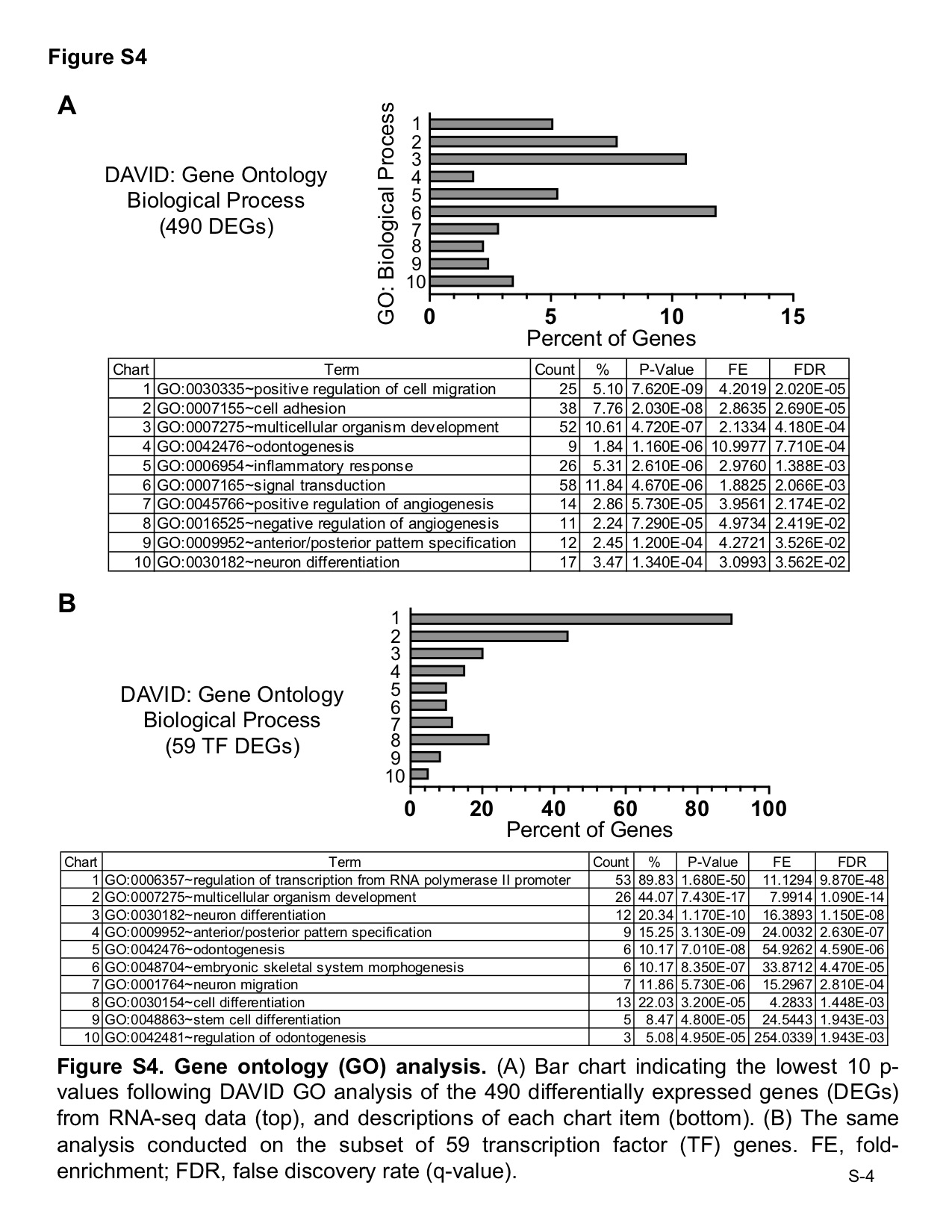

Supplement: Supplemental Material [file KCBT_A_2271638_SM4140.zip › Streitfeld_Fig S4_REVISED.jpg]

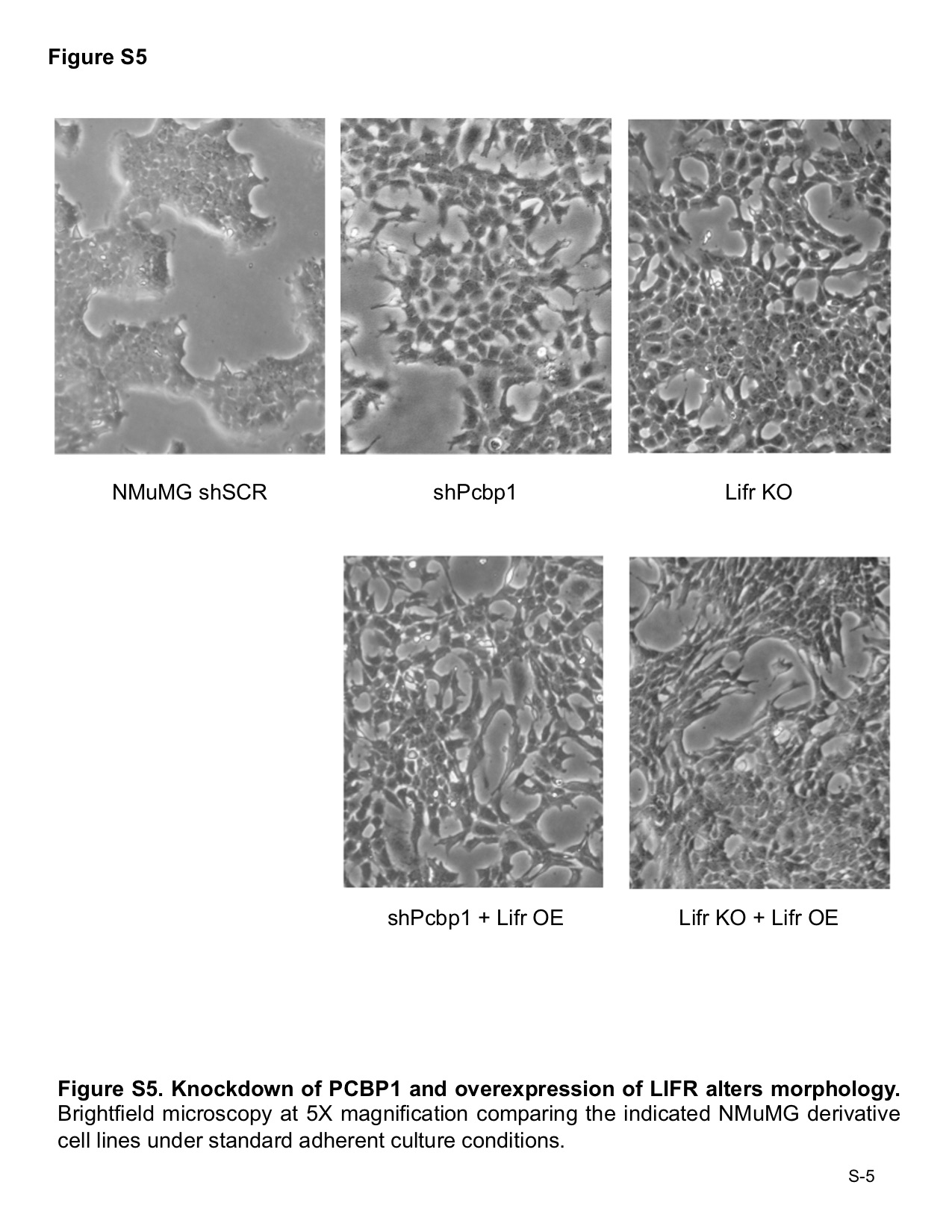

Supplement: Supplemental Material [file KCBT_A_2271638_SM4140.zip › Streitfeld_Fig S5_REVISED.jpg]

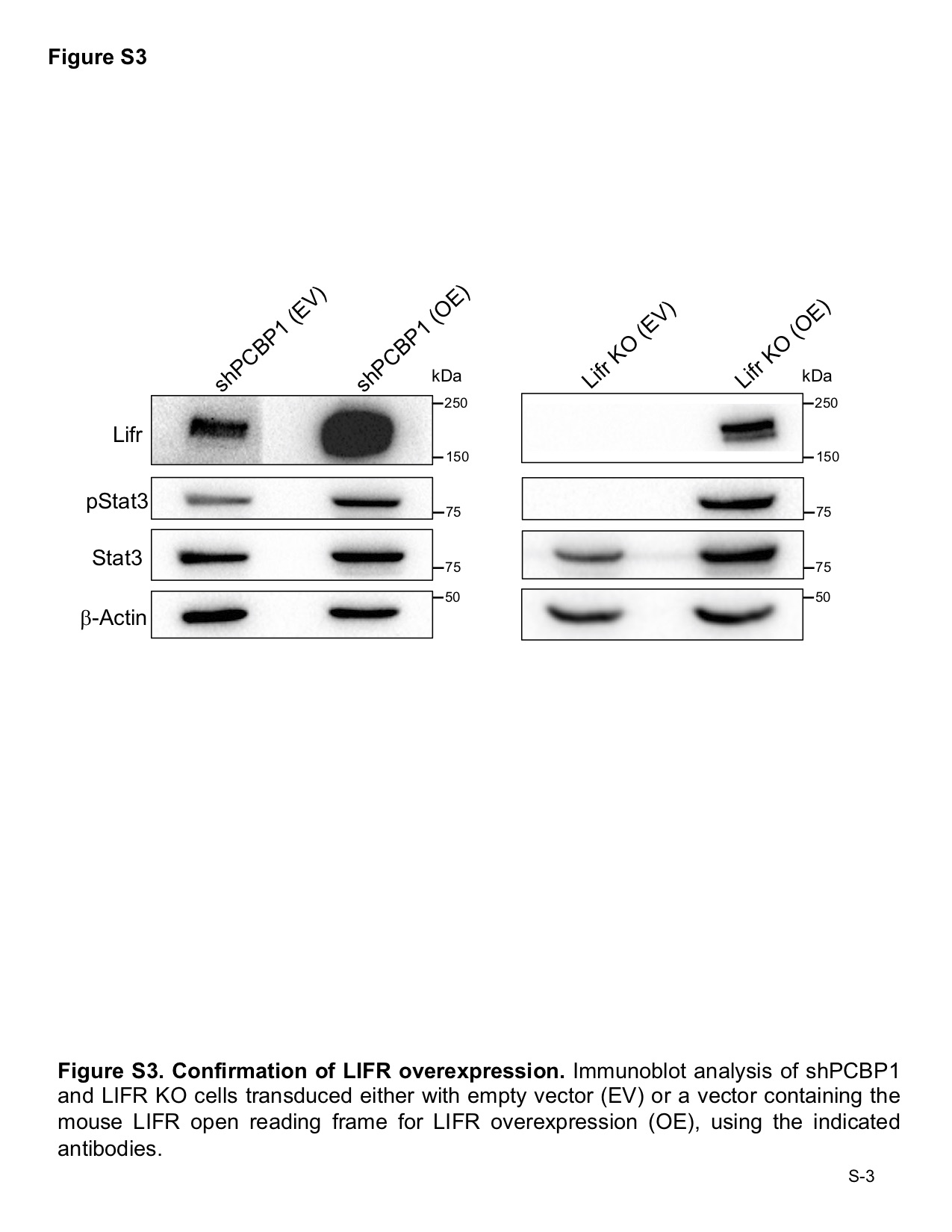

Supplement: Supplemental Material [file KCBT_A_2271638_SM4140.zip › Supplementary Figures S3 (FINAL).jpg]
